# Supplementary material for: Dual function of OmpM as outer membrane tether and nutrient uptake channel in diderm Firmicutes
Source: Nat Commun. 2023 Nov 6;14:7152. doi: 10.1038/s41467-023-42601-y (PMC10628300; doi:10.1038/s41467-023-42601-y)
Supplement: Supplementary file 1 — Supplementary Information [file 41467_2023_42601_MOESM1_ESM.pdf]

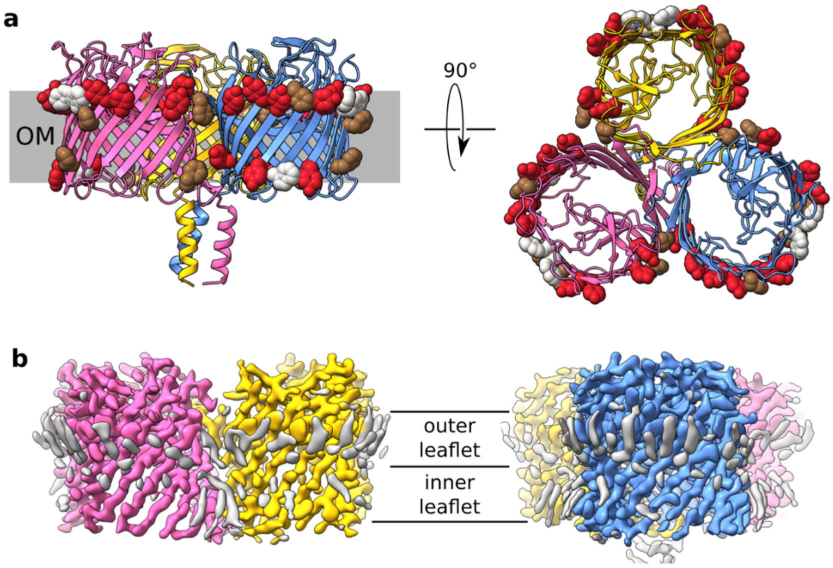

Supplementary Figure 1. VpOmpM1 aromatic girdle and putative lipid density. a Aromatic residues forming the aromatic girdle shown in space-filling representation on the cartoon of C1 VpOmpM1 reconstruction. Brown, phenylalanine; red, tyrosine; grey, tryptophan. **b** Cryo-EM density of the C3 VpOmpM1 reconstruction. Protein density coloured by chain; grey, putative lipid or detergent density.

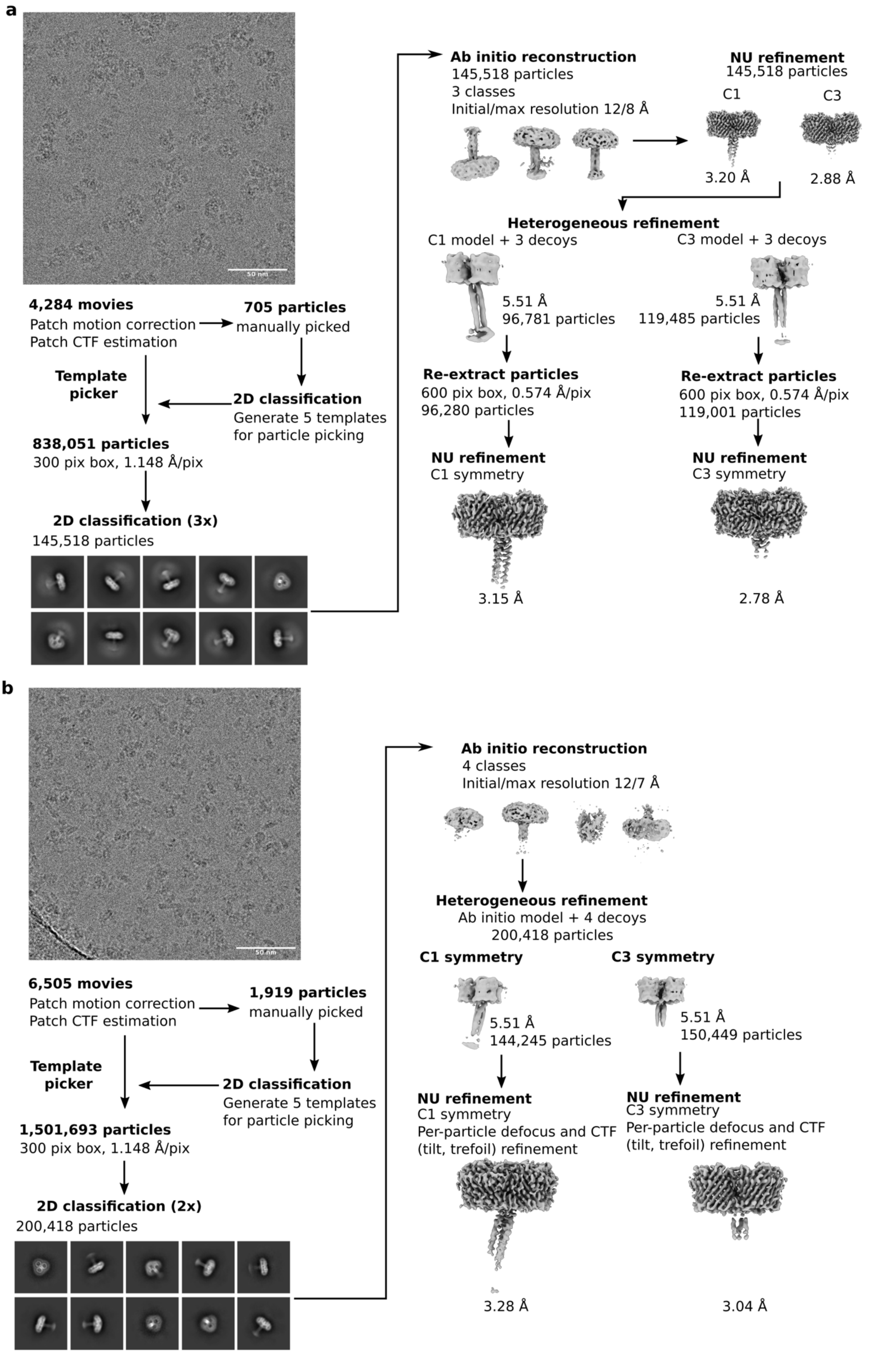

Supplementary Figure 2. Cryo-EM data processing. Cryo-EM data processing workflows for VpOmpM1 expressed in *E. coli* (**a**) and purified from *V. parvula* (**b**), showing representative motion-corrected movies, 2D class averages, and intermediate and final cryo-EM maps. A total of 4,284 and 6,505 movies were collected of grids with VpOmpM1 expressed in *E. coli* and *V. parvula*, respectively.

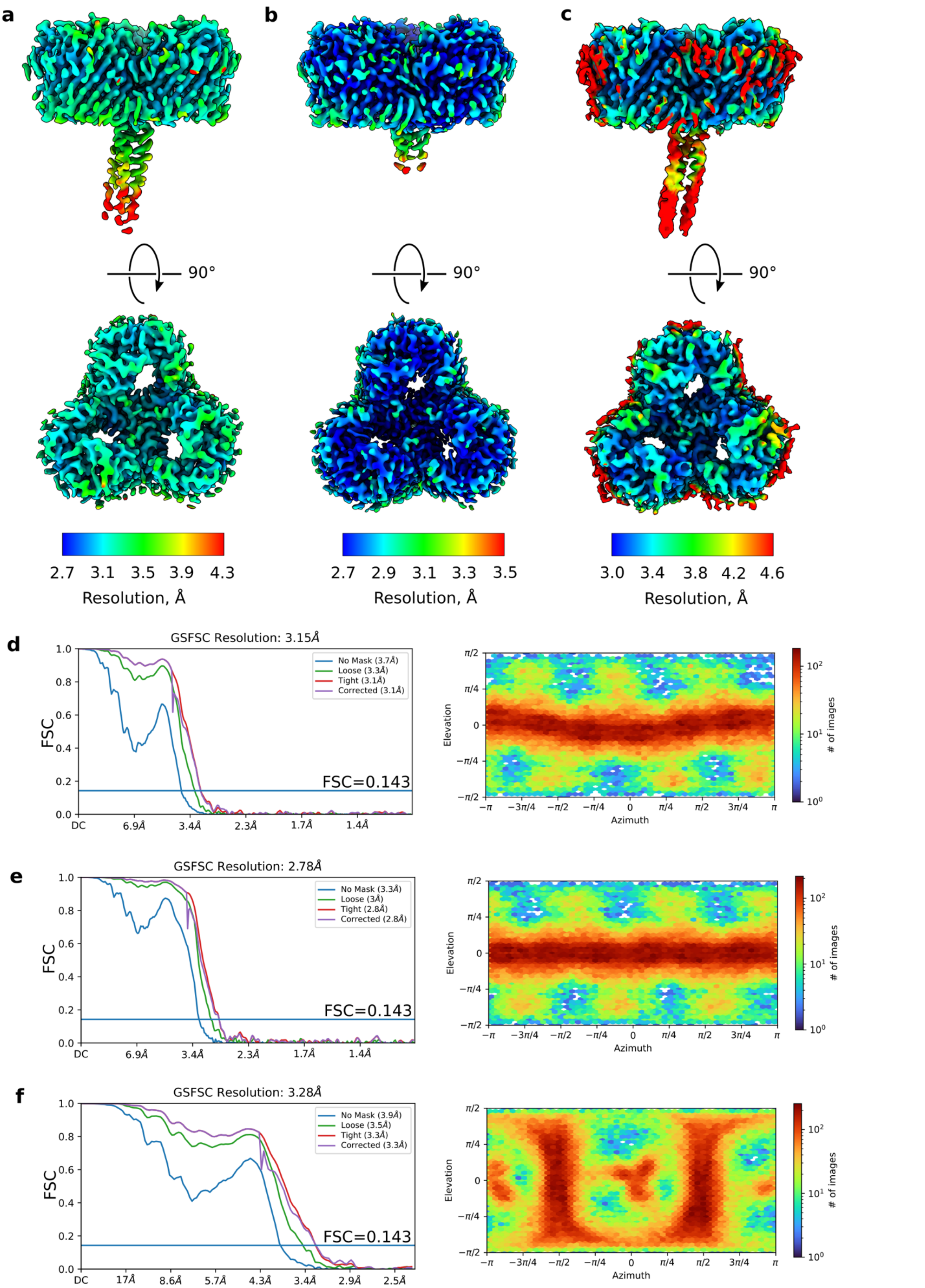

Supplementary Figure 3. Cryo-EM map local resolution estimates, FSC curves, and angular distribution plots. **a**, **d** VpOmpM1 purified from *E. coli*, C1 symmetry. **b**, **e** VpOmpM1 purified from *E. coli*, C3 symmetry. **c**, **f** VpOmpM1 purified from *V. parvula*, C1 symmetry.

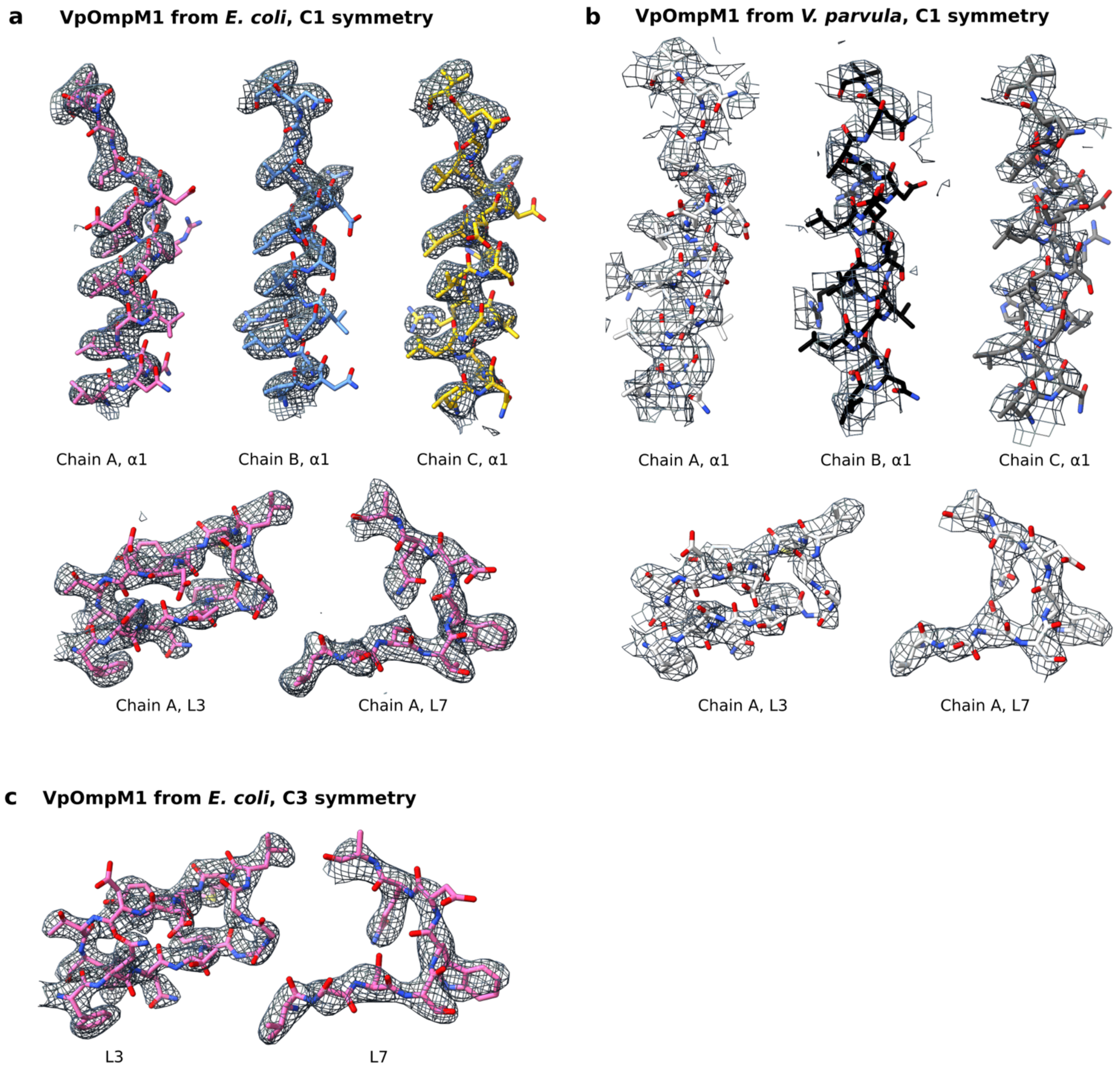

Supplementary Figure 4. Cryo-EM representative map-to-model fits. Densities and models for the stalk coiled-coil helix for each protomer and the eyelet loops 3 and 7 from a single protomer are shown for VpOmpM1 purified from *E. coli* (**a**) and from*V. parvula* (**b**) in C1 symmetry. Only the eyelet model fit for VpOmpM1 purified from *E. coli* with applied C3 symmetry is shown (**c**), as it was not possible to build a model into the stalk density in the symmetrized map. Protein models are shown in stick representation. Cryo-EM density is represented by a mesh.

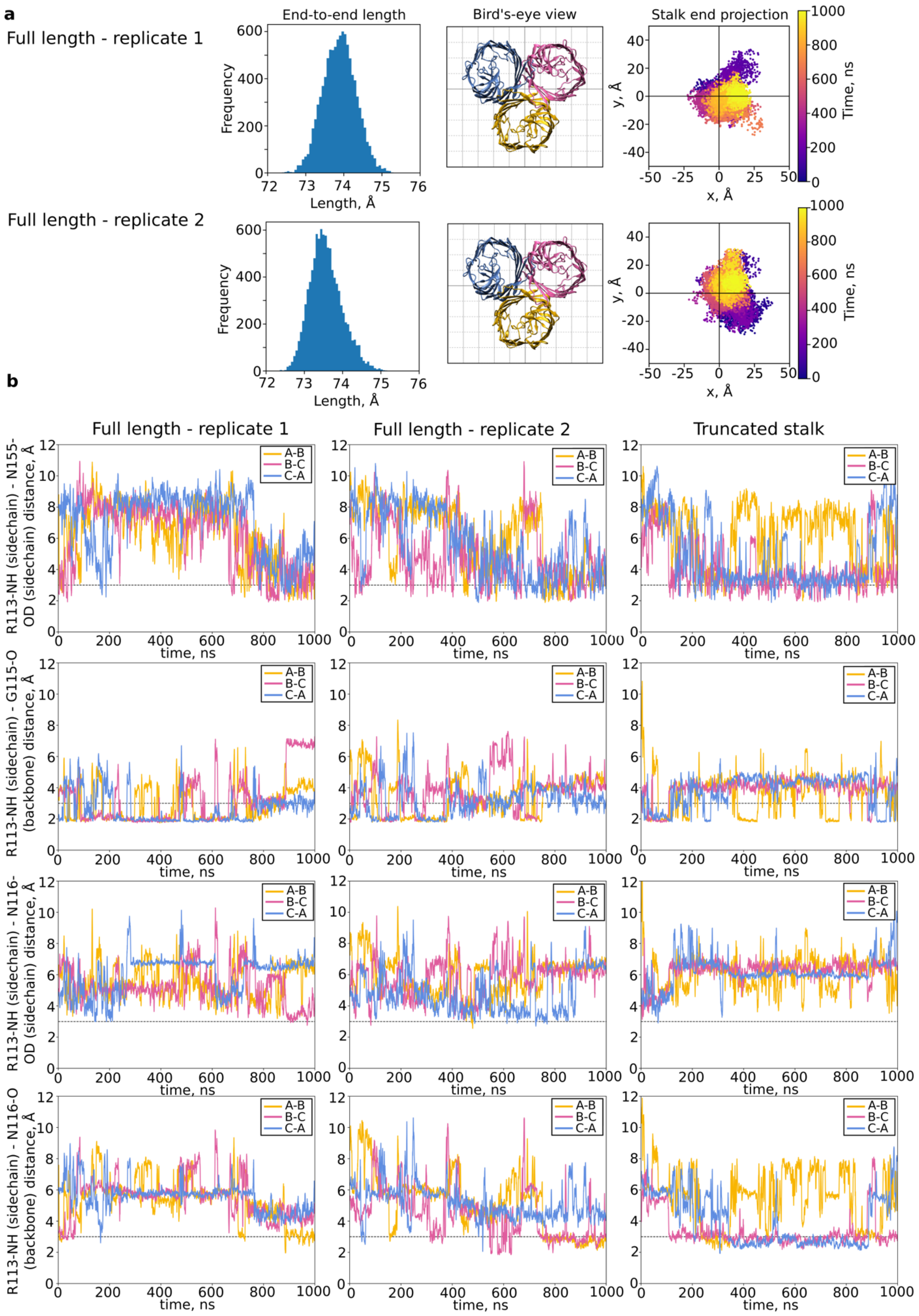

Supplementary Figure 5. All-atom molecular dynamics simulation replicates. **a** Stalk length and end projection plots for the two replicates of all-atom simulations with the native VpOmpM1 and AlphaFold2 graft model (full-length). The bird's-eye view coordinates correspond to the plot coordinates in the stalk projection plot. **b** Hydrogen bond distances throughout the simulation. The 'truncated stalk' simulation was performed with the native VpOmpM1 model without the AlphaFold2 graft. Data for replicate 1 shown in this figure are the same as the data shown in Figure 2.

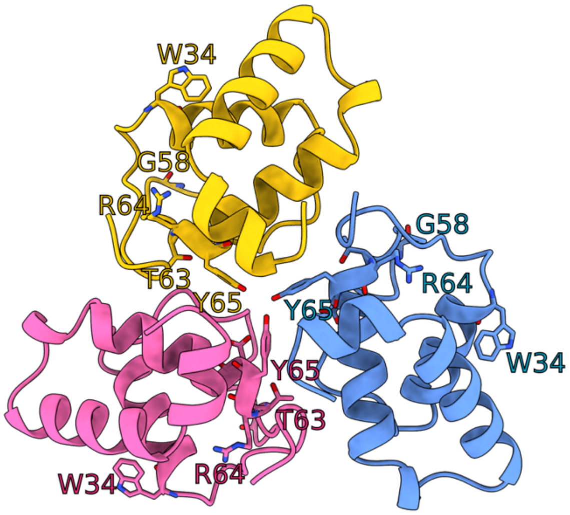

Supplementary Figure 6. Putative PG-binding motifs in the SLH crystal structure. Residues that are part of motifs conserved in SLH domains are shown in stick representation. Motif residues are not located in intra-protomer grooves as seen in crystal structures of SLH domains from monoderm Firmicutes and in the extended conformation of the VpOmpM1 stalk predicted by AlphaFold2 (Figure 3).

Rank	Chain	Z	rmsd	lali	nres	%id	Description
1	5f7l-C	5.7	2.5	57	375	11	Blood group antigen binding adhesin BabA
2	6bml-B	5.2	3.3	67	294	12	Human palmitoyltransferase DHHC20
3	6fws-B	5.2	3.3	68	683	4	Helicase DinG
4	6gmm-A	5.2	2.9	61	433	11	Adhesin LabA
5	6fws-A	5.0	2.7	65	686	8	Helicase DinG
6	7khm-A	4.8	3.3	68	290	12	Human palmitoyltransferase DHHC20
7	5f7w-A	4.8	2.7	60	419	10	Blood group antigen binding adhesin BabA
8	5f7y-A	4.8	3.2	62	417	10	Blood group antigen binding adhesin BabA
9	5has-A	4.7	3.2	64	381	5	DCB-HUS domain of Sec7
10	5f8q-A	4.7	2.4	56	419	11	Blood group antigen binding adhesin BabA

Supplementary Figure 7. SLH domain crystal structure DALI search results. DALI[1] analysis shows that the VpOmpM1 SLH domain crystal structure (PDB 8BZ2) has low similarity to other proteins from the PDB.

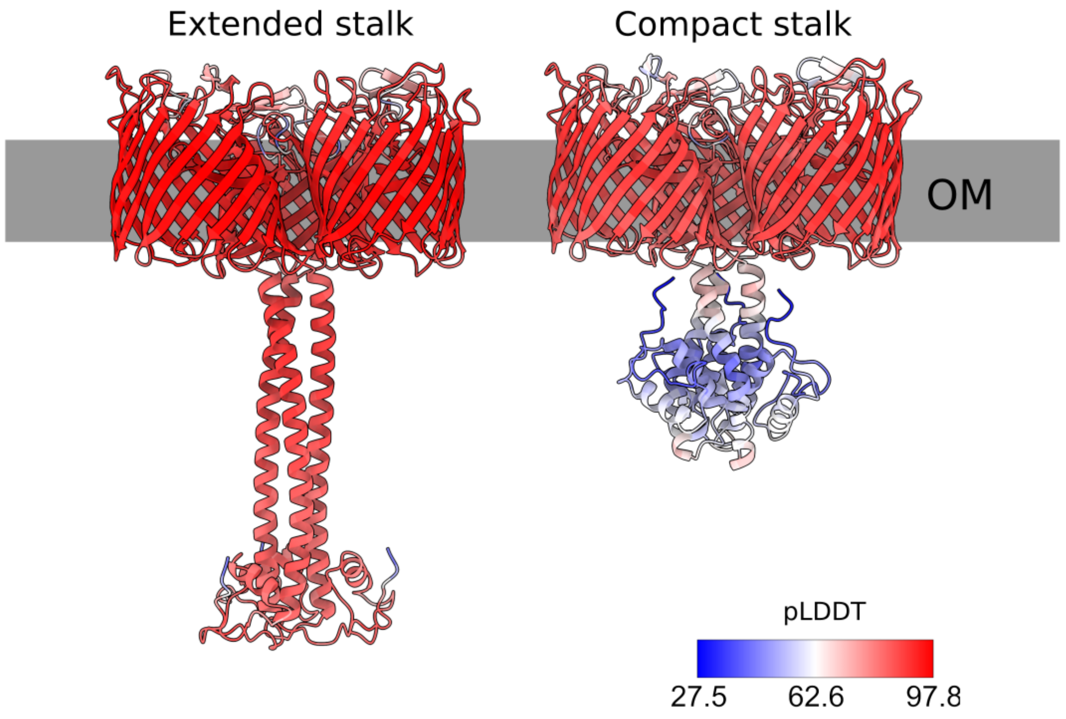

Supplementary Figure 8. VpOmpM1 trimer AlphaFold2[2] predictions. Cartoons coloured by per residue confidence score (colour key): pLDDT>90 – very high; 90>pLDDT>70 confident; 70>pLDDT>50 low; pLDDT<50 – very low.

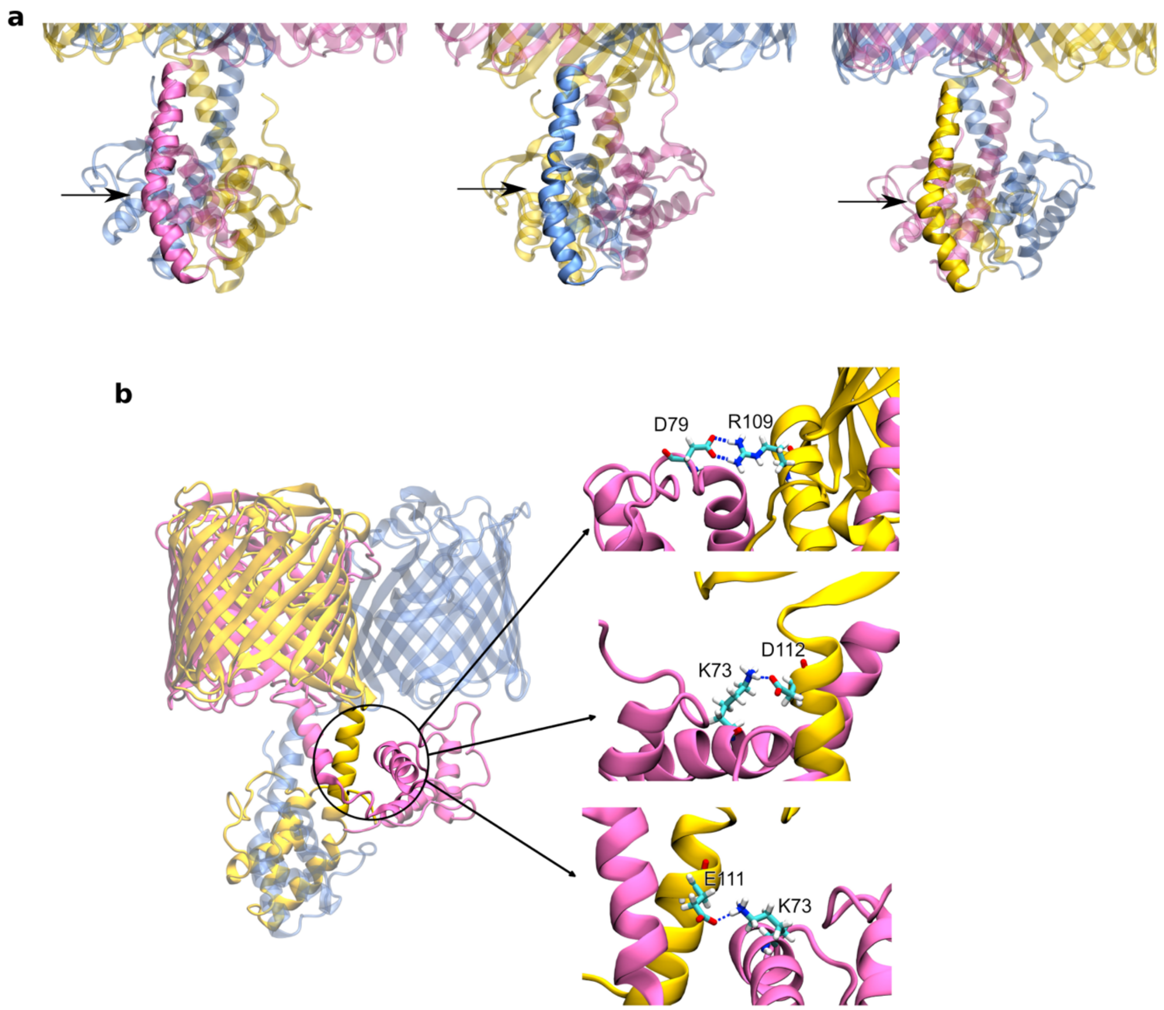

Supplementary Figure 9. VpOmpM1 compact stalk simulation. **a** The stalk α–helices in each protomer are kinked at residues 97-102 (arrows). This kink is the hinge that allows movement of the stalk towards the OM, as seen for the pink protomer in the simulation. **b** The conformation of the pink protomer is stabilised by salt bridge formation with the stalk of the yellow protomer. Salt bridge occupancy throughout the simulation (top to bottom): 14.33%, 3.21% and 1.27%.

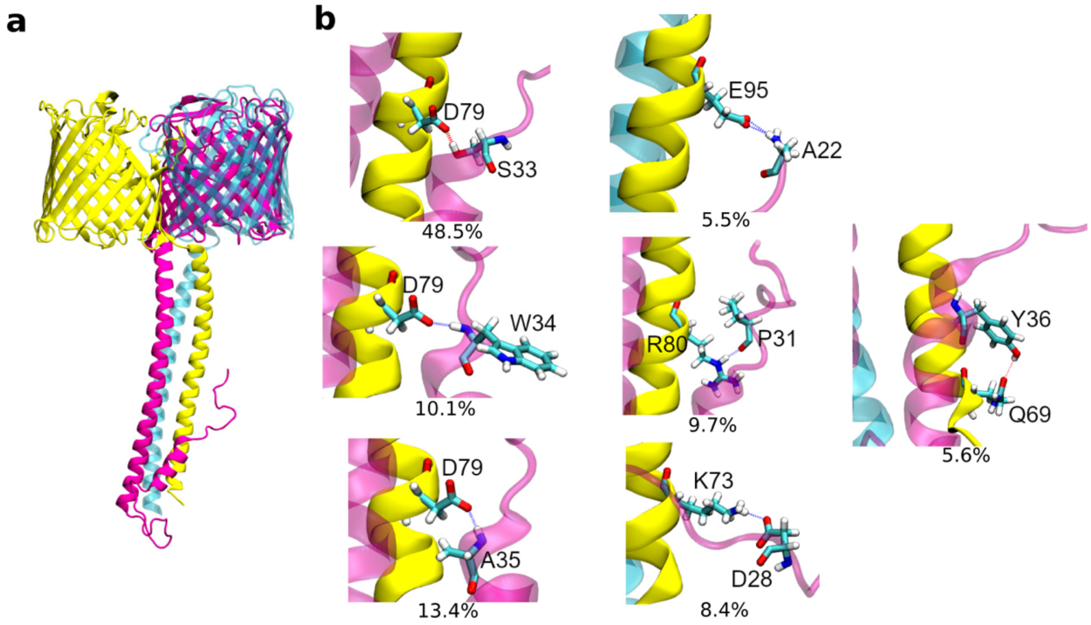

Supplementary Figure 10. Interaction of SLH domain with coiled-coil during extended stalk MD simulations. a During one all-atom simulation of VpOmpM1 with the grafted stalk, the SLH domain of one protomer (pink) unfolded and formed contacts with the coiled-coil region of another protomer (yellow). The SLH domains of the yellow and cyan protomers are not shown for clarity. **b** Close-up views of the SLH-coiled coil interactions. The percentage occupancy of each interaction throughout the 1 μs simulation is shown under each panel.

a

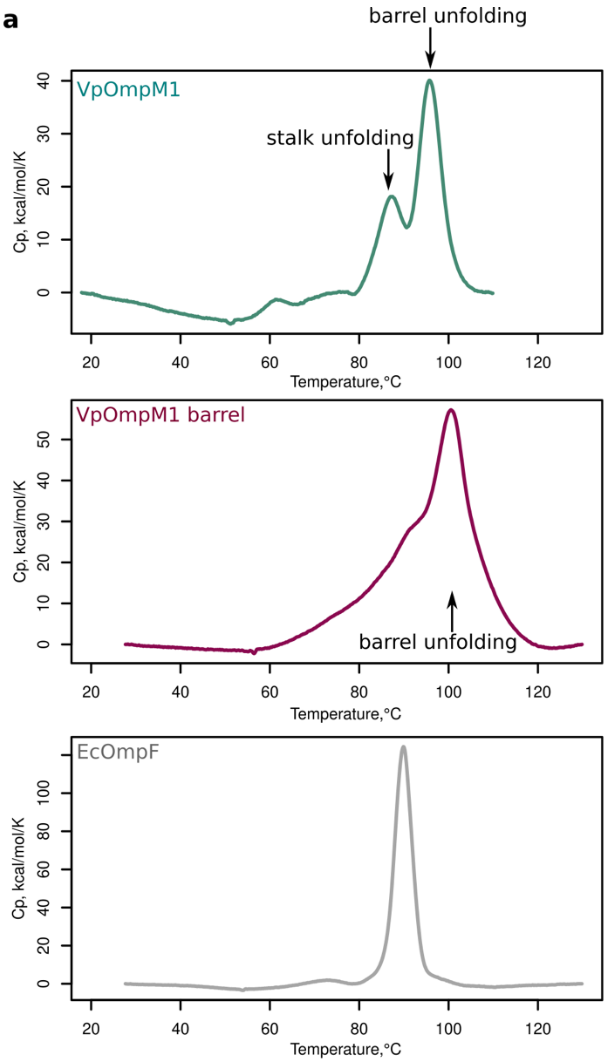

b

Sample	Melting temperature	
	T_m1	T_m2
VpOmpM1	87.46°C	95.74°C
VpOmpM1 barrel	92.27°C	101.06°C
EcOmpF	89.93°C	N/A

Supplementary Figure 11. Protein melting temperature analysis. **a** Dynamic scanning calorimetry thermograms for full-length VpOmpM1, VpOmpM1 barrel domain, and EcOmpF. Measurements were performed on a Malvern VP Capillary DSC instrument, using 18.7 μM protein in 10 mM HEPES-NaOH pH 7.0, 100 mM NaCl and 0.12% DM in each experiment. **b** Data were fitted and the melting temperatures (T_m) were extracted via the instrument manufacturer's software.

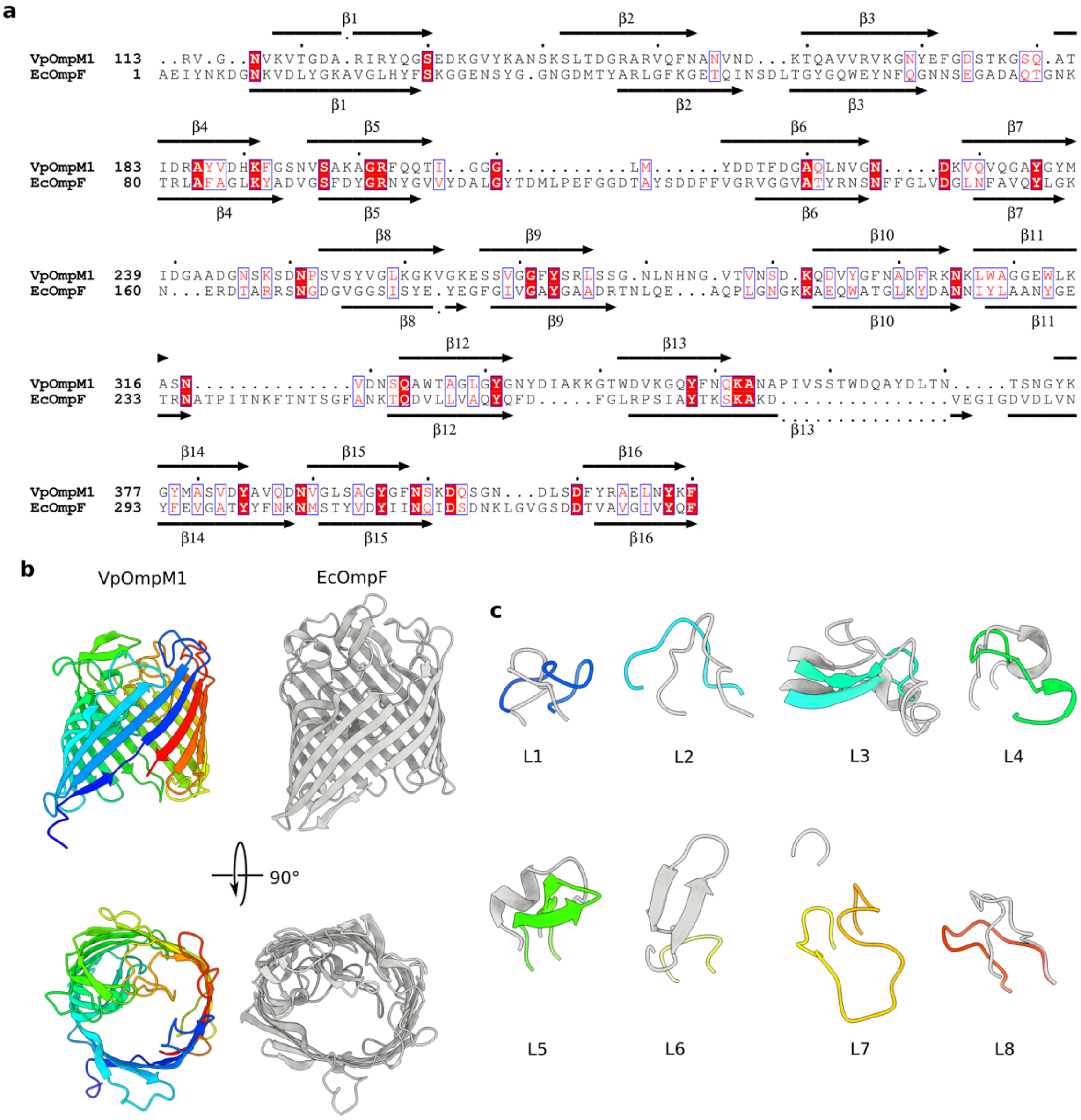

Supplementary Figure 12. Comparison of VpOmpM1 and EcOmpF. **a** Alignment of VpOmpM1 β-barrel and EcOmpF amino acid sequences. The alignment was generated by sequence-independent pairwise structure alignment of VpOmpM1 and EcOmpF (PDB 2ZFG) using TM-align[3] (RMSD 3.84 Å). Residues forming the barrel β-strands are annotated above and below the alignment for VpOmpM1 and EcOmpF, respectively. The sequences share 8% identity and 25% similarity. **b** Comparison of the VpOmpM1 β-barrel from C3 reconstruction (rainbow, N-terminus blue, C-terminus red) and EcOmpF (2ZFG; grey) structures. Views generated from a superposition. **c** Comparison of the extracellular loops (L1-8) of the two β-barrels as viewed from inside the β-barrel.

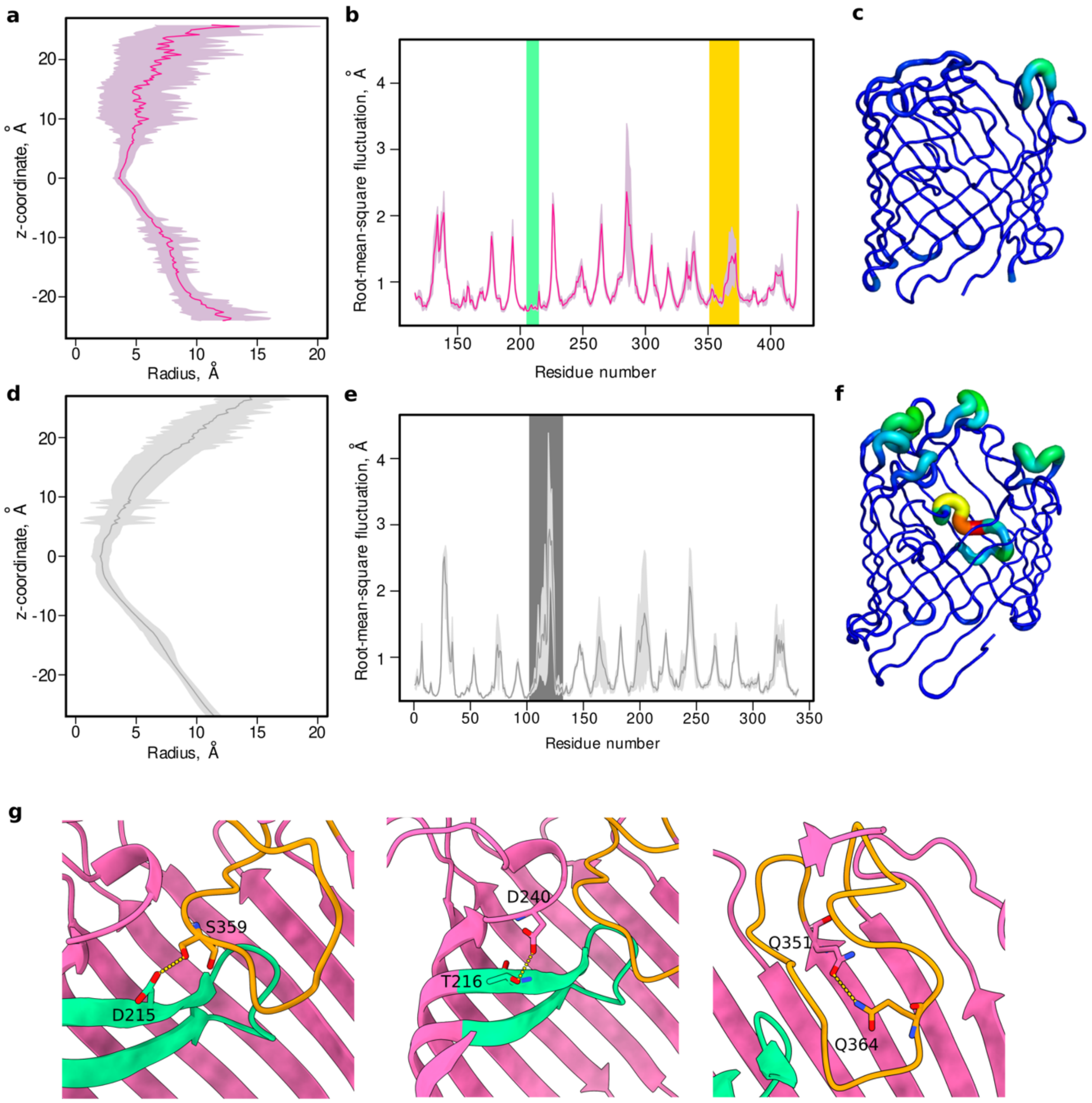

Supplementary Figure 13. Fluctuations in the channel regions of VpOmpM1 and EcOmpF during MD simulations. **a-f** Analysis of residue fluctuations in VpOmpM1 and EcOmpF during MD simulations. HOLE[4] analyses of VpOmpM1 (**a**) and EcOmpF (**d**) calculated averaged from all-atom MD simulation frames: 1 frame every 10 ns over the final 100 ns from 3 simulations, averaged over the 3 protomers (VpOmpM1, n=90 traces); 1 frame every 10 ns over the final 300 ns of a single all-atom simulation, averaged over the three protomers (EcOmpF, n=90 traces). Lines show the average values ± S.D. depicted by the shaded area. **b, e** Per-residue root-mean-square fluctuation (RMSF) plots for VpOmpM1 (**b**) and EcOmpF (**e**) during MD simulations. Same sampling as in **a** and **d**. Lines show the average values ± S.D. depicted by the shaded area. Residues forming the constriction loops are highlighted: VpOmpM1 L3, green; VpOmpM1 L7, orange; EcOmpF L3, grey. **c, f** Per-residue RMSF values converted to B-factors and displayed on VpOmpM1 (**c**) and EcOmpF (**f**) structures on the same scale. Cartoon thickness and colour (blue to red) are proportional to B-factor value. **g** Examples of interactions observed during simulations that likely restrict movement of the eyelet-forming loops of VpOmpM1: between the L3 and L7 loops (left), between L3 and L4 (middle), and between L7 and the internal barrel wall (right).

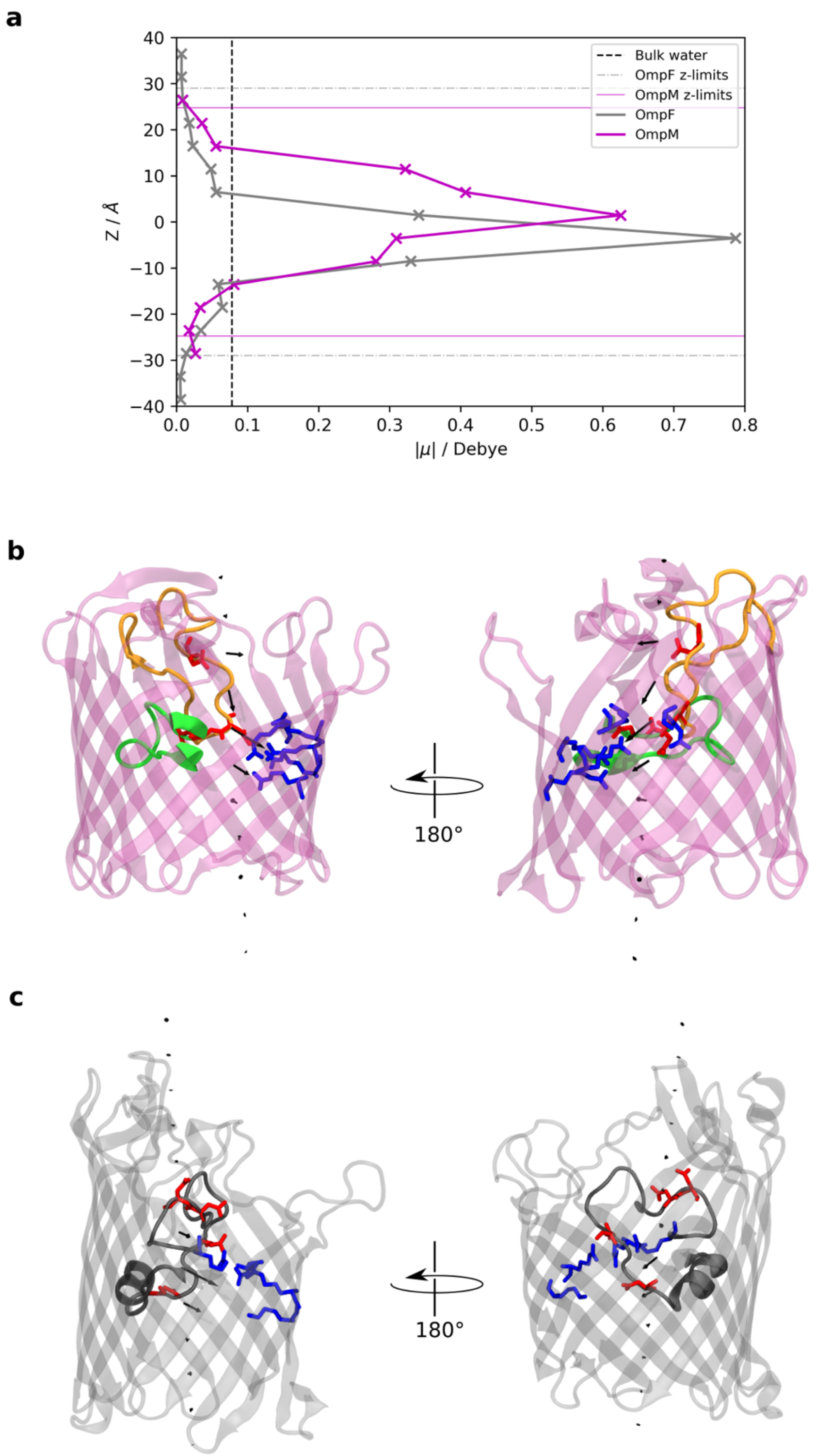

Supplementary Figure 14. Transverse electric field properties in VpOmpM1 and EcOmpF channels. **a** Total water dipole moment by z-coordinate, normalised to the number of water molecules in each z-slice. **b, c** Arrows indicate the direction of the dipole moment averaged across the waters in a particular z-slice over the final 100 ns of equilibrium simulations for VpOmpM1 (**b**) and EcOmpF (**c**). The length of the arrow is proportional to the magnitude of the dipole moment. VpOmpM1 loop 3 is green and loop 7 is orange; EcOmpF loop 3 is black. Basic loop residues are in blue and acidic residues are in red.

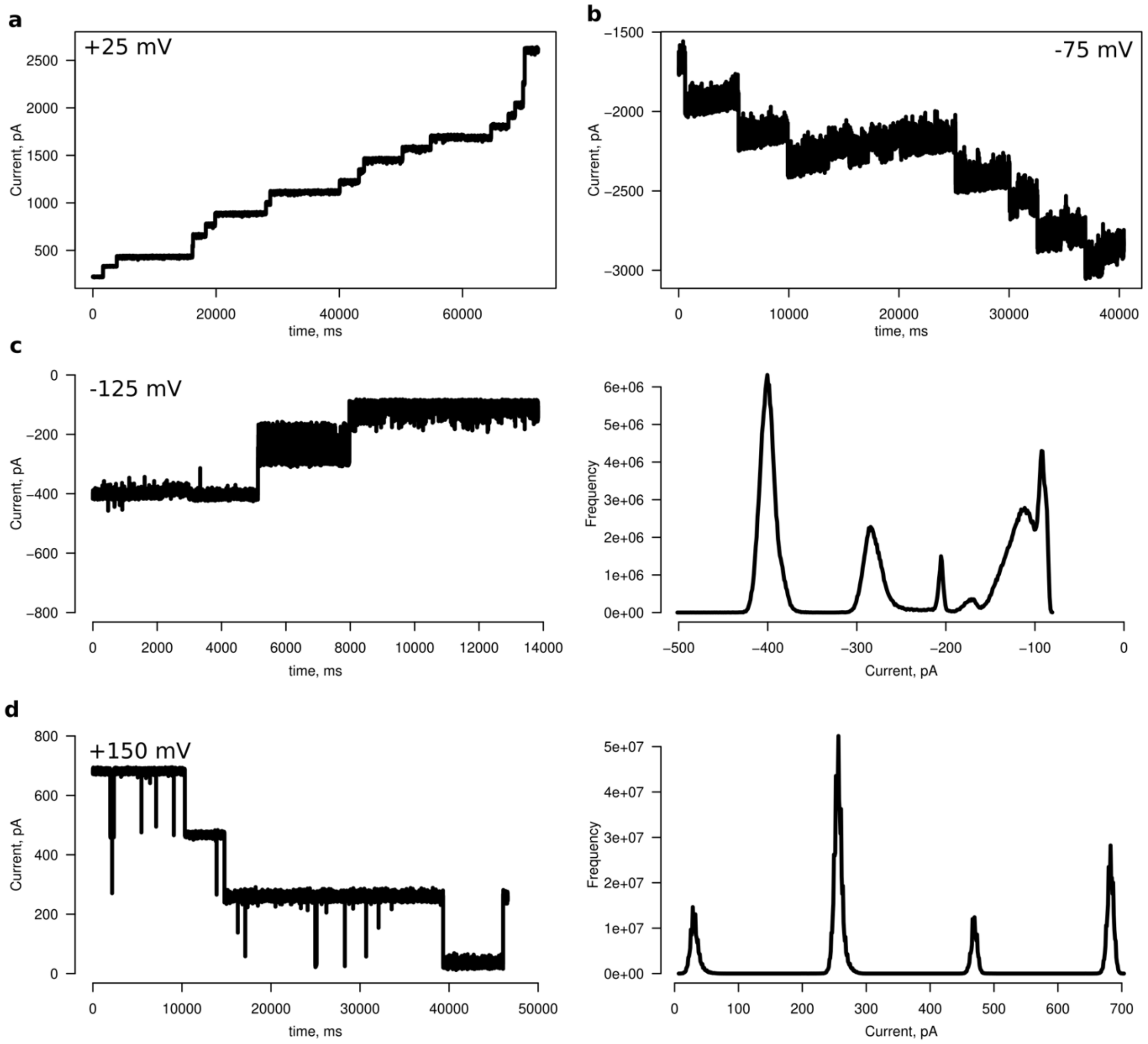

Supplementary Figure 15. Representative electrophysiology recordings. **a, b** Representative ion-current traces of full-length VpOmpM1 (**a**) and the barrel-only construct (**b**) showing multiple channel insertion events observed as 'steps' or sudden increases in current. **c, d** Ion-current traces for full-length VpOmpM1 (**c**) and the barrel-only construct (**d**) showing channel gating activity at high potential (left), with the corresponding all-current point histograms shown on the right. A 2.5 kHz low-pass eight-pole Bessel filter was applied to all traces. The applied voltage is indicated on each trace.

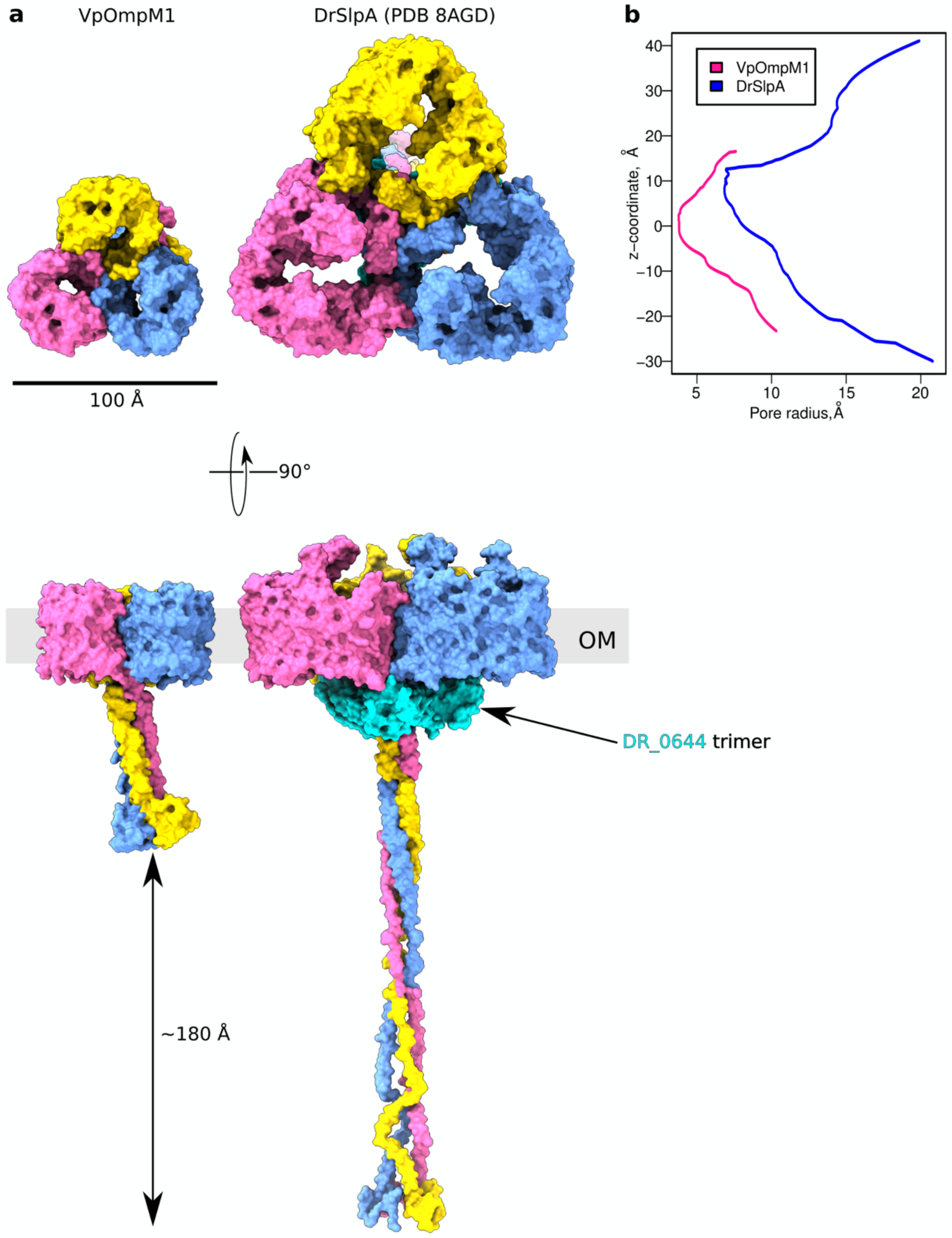

Supplementary Figure 16. Comparison of VpOmpM1 and DrSlpA structures. **a** To-scale surface representations of VpOmpM1 and *Deinococcus radiodurans* SlpA (DrSlpA) (PDB 8AGD[5]) from outside the cell (top) and from the OM plane (bottom). **b** HOLE[4] profiles of VpOmpM1 and DrSlpA. Note that the profiles show the path of a ball-shaped probe via the narrowest part of the pore and, therefore, this analysis grossly underestimates the size of the SlpA pore, since the constriction is not roughly circular as in VpOmpM1.

Supplementary Table 1. Cryo-EM data collection, processing and refinement statistics.

	**VpOmpM1 from *E. coli***		**VpOmpM1 from *V. parvula***
**Data collection**			
Electron microscope	FEI Glacios		FEI Glacios
Voltage (kV)	200		200
Spherical aberration (µm)	2.7		2.7
Camera	Falcon 4 (counting)		Falcon 4 (counting)
Energy filter	none		none
Magnification	240,000		240,000
Pixel size (Å)	0.574		0.574
Total dose (e^-/Å^2)	50.1		50
Defocus minimum maximum (µm)	-1.0 to -2.0		-0.8 to -2.0
Number of movies collected	4,284		6,505
**Image Processing**			
Imposed symmetry	C1	C3	C1
Initial number of particles	838,051	838,051	1,501,693
Final number of particles	96,280	119,001	144,245
Global resolution (FSC = 0.143)	3.15	2.78	3.28
Map sharpening B-factor (Å^2)	-70.2	-85.5	-97.5
**Refinement**			
Model composition			
Non-hydrogen atoms	7,386	7,077	7,406
Protein residues	963	924	968
R.m.s. deviations			
Bonds lengths (Å)	0.003	0.002	0.004
Bond angles (˚)	0.493	0.420	0.563
Validation			
MolProbity score	1.56	1.39	1.76
Clash score	4.52	3.57	6.45
Rotamer outliers (%)	0	0	0
Ramachandran plot			
Favoured (%)	95.30	96.41	93.97
Outliers (%)	0	0	0
PDB	8BYM	8BYT	8BYS
EMDB	EMD-16328	EMD-16333	EMD-16332

Supplementary Table 2. Crystallography data collection, processing and refinement parameters.

	**VparOmpM1 stalk***
**Data collection**	
DLS beamline	I03
Wavelength	0.89842
Space Group	H3
Unit cell parameters	
a, b, c (Å)	118.71, 118.71, 47.24
α, β, γ (°)	90, 90, 120
Molecules in AU	3
Resolution range (Å)	34.78-1.57 (1.57-1.6)
$I/\sigma I$	17.1 (0.7)
Completeness (%)	99.5 (92.9)
Multiplicity	9.0 (4.7)
Rpim (%)	1.7 (88.4)
$CC_{1/2}$ (%)	100 (23)
**Phasing**	
Ab initio	Arcimboldo[6]
**Refinement**	
Resolution (Å)	34.78-1.7
Rwork/Rfree (%)	21.9 /24.8
Reflections	
Non-hydrogen atoms	2,015
Protein only	1,918
Average B-factor (Å^2)	39.68
Rmsd	
Bond lengths (Å)	0.011
Bond angles (°)	1.21
MolProbity clashscore	3.75
Ramachandran plot	
Favoured (%)	99.59
Outliers (%)	0
PDB	8BZ2

*Statistics for the highest-resolution shell are shown in parentheses.

Supplementary Table 3. Bacterial strains used in this study.

Strain	Genotype	Reference or Source
E. coli **DH5α**	*F- endA1 glnV44 thi-1 recA1 relA1 gyrA96 deoR nupG purB20* φ80d*lacZ*ΔM15 Δ(*lacZYA-argF*)U169, hsdR17($r_K^- m_K^+$), λ^-	Promega
E. coli **TOP10**	*F- mcrA Δ(mrr-hsdRMS-mcrBC) φ80lacZΔM15 ΔlacX74 nupG recA1 araD139 Δ(ara-leu)7697 galE15 galK16 rpsL(StrR) endA1 λ-*	Invitrogen
E. coli **BL21(DE3)**	*F- ompT lon hsdSB (rB- mB-) gal dcm* (DE3)	Invitrogen
E. coli **C43(DE3) *ΔcyoABCD***	*F- ompT lon hsdSB (rB- mB-) gal dcm* (DE3) *lacUV5*** *ΔcyoABCD*	[7] (modified)
E. coli **MFDpir**	*MG1655 RP4-2-Tc ::[Δmu1 ::aac(3)IV-ΔaphA-Δnic35-Δmu2 ::zeo] ΔdapA :erm-pir) ΔrecA*	[8]
V. parvula **SKV38**	Wild type strain	[9]
V. parvula **SKV38 Δ*ompM1-3***	*ΔFNLLGLLA_01389-7::tetM*	[10]

Supplementary Table 4. Plasmids used in this study.

Plasmid	Description	Reference or Source
**pB22**	pBAD22 vector with *E. coli* TamA signal sequence, N-terminal His_7-tag	[11,12]
**pJW45**	pB22 with beta barrel of *ompM1* cloned between XhoI and XbaI restriction sites	This study
**pJW46**	pB22 with *ompM1* (without signal peptide) CDS cloned between XhoI and XbaI restriction sites	This study
**pJW48**	pRPF185 with His-tagged *ompM1* (native RBS) cloned into the *Sac*I restriction site	This study
**pRPF185**	*Escherichia/Clostridium* shuttle conjugative expression vector with *tet* promoter	[13]
**pBAD24**	Vector for expressing proteins with their native signal sequence in *E. coli*	[11]
**pBAD24-EcOmpF**	pBAD24 vector with the full-length *E. coli* OmpF coding sequence cloned between EcoRI and XbaI restriction sites	This study
**pET28b**	*E. coli* expression vector	Novagen
**pET28b-SLH_22-107**	pET28b with *ompM1* SLH domain and stalk residues 22-107 coding region cloned between NcoI and XhoI sites (C-terminal His_6-tag)	This study
**pB22-00518_21-200**	pB22 with beta barrel (residues 21-200) of *FNLLGLLA_00528* cloned between XhoI and XbaI restriction sites	This study
**pB22-00833**	pB22 with beta barrel of *FNLLGLLA_00833* cloned between XhoI and XbaI restriction sites	This study

Supplementary Table 5. Primers* used in this study.

Primer	Sequence	Description
**JW172**	agcgttaacagatctgagcttaagaaagaaggaattcattatgaaaaaac	Forward primer for amplifying full length *ompM1* with its native RBS with homology arm to the pRPF185 digested with SacI
**JW201**	ccatcaccatcaccatcacctcgagcgcttggaagaccgtgtag	Forward primer for amplifying OmpM1 beta barrel with homology arm to the pB22 digested with XhoI
**JW202**	ctgaagcgggtgtgaatactctagattagaatttgtaatttaattctgcacg	Reverse primer for amplifying *ompM1* with homology arm to the pB22 digested with XbaI
**JW203**	ccatcaccatcaccatcacctcgaggctgcaaatccattctcc	Forward primer for amplifying *ompM1* without signal peptide with homology arm to the pB22 digested with XhoI
**JW206**	tctcctttactgcaggagct**ttaatgatgatgatgatgatgaccacc**gaatttgtaatttaattctgcacggtag	Reverse primer for amplifying *ompM1*, adding C-terminal 6His tag (indicated in bold) with homology arm to the pRPF185 digested with SacI.
**00833_F**	atactcgaggcatttgctgcagctcc	Forward primer for amplifying *FNLLGLLA_00833* without signal peptide with XhoI site
**00833_R**	atatctagattagaaggagtagcctacacc	Reverse primer for amplifying *FNLLGLLA_00833* without signal peptide with XbaI site
**00518_F**	atactcgagactcctcaaactcaattcaataaag	Forward primer for amplifying *FNLLGLLA_00518* barrel with XhoI site
**00518_R**	atatctagattaaccaccgaaacggtaagataatc	Reverse primer for amplifying *FNLLGLLA_00518* barrel with XbaI site
**stalk_F**	ataccatggctgcaaatccattctccg	Forward primer for amplifying *ompM1* stalk region with NcoI site
**stalk_R**	atactcgagctttacattacctacacggtc	Reverse primer for amplifying *ompM1* stalk region with XhoI site

* The part of the primer hybridizing to the template is indicated by an underscore.

Supplementary Table 6. Position restraints, timesteps (dt), and durations of the equilibration phases in the all-atom MD simulations.

Equilibration phase	Position restraint / kJ mol^{-1} nm^{-2}				dt/ fs	Duration / ns
	Protein Backbone	Protein Sidechains	Lipid Headgroups	Dihedrals		
**NVT1**	4000	2000	1000	1000	1	0.125
**NVT2**	2000	1000	400	400	1	0.125
**NPT1**	1000	500	400	200	2	0.5
**NPT2**	500	200	200	200	2	0.5
**NPT3**	200	50	40	100	2	0.5
**NPT4**	50	-	-	-	2	0.5

Supplementary Table 7. VpOmpM1 simulation composition.

System	Contents									Total atoms	Box dimensions, nm
	OmpM protomers	POPE	POPG	Cardiolipin	LPS	Potassium	Chloride	Calcium	TIP3P Water		
**Truncated stalk (residue 100 onwards)**	3	180	10	10	62	258	178	279	69,146	317,563	12.74 x 12.74 x 21.56
**Full length extended stalk**	3	162	9	9	55	324	241	247	91,443	377,626	12.22 x 12.22 x 27.40
**Full length compacted stalk**	3	162	9	9	56	268	185	252	71,804	319,677	12.25 x 12.25 x 23.18

Supplementary Table 8. EcOmpF simulation composition.

System	Contents									Total atoms	Box dimensions, nm
	OmpF protomers	POPE	POPG	Cardiolipin	LPS	Potassium	Chloride	Calcium	TIP3P Water		
**EcOmpF**	3	180	10	10	63	273	143	283	56,781	282,355	12.71 x 12.71 x 19.61

Supplementary Table 9. Substrate concentrations used in liposome swelling assays.

Substrate	Concentration (mM)
**Figure 6** – VpOmpM1, VpOmpM1 barrel and EcOmpF	
**Lactate**	8
**Acetate**	8
**Putrescine**	8
**Arginine**	8
**Lysine**	8
**Glutamate**	10
**Aspartate**	8
**Glycine**	15
**Alanine**	15
**Leucine**	12
**Methionine**	15
**Arabinose**	12
**Glucose**	12
**Fructose**	12
**Lactose**	15
**Maltose**	15
**Sucrose**	15
**Kanamycin**	8
**Ampicillin**	8
**Gentamicin**	8
**Figure 7** – FNLLGLLA_00518 barrel, VpOmpM1 barrel and EcOmpF	
**Lactate**	10
**Putrescine**	8
**Glycine**	15
**Arabinose**	10

Supplementary references

1. Holm, L. Dali server: structural unification of protein families. *Nucleic Acids Res.* **50**, W210–W215 (2022).
2. Jumper, J. *et al.* Highly accurate protein structure prediction with AlphaFold. *Nat. 2021 5967873* **596**, 583–589 (2021).
3. Zhang, Y. & Skolnick, J. TM-align: a protein structure alignment algorithm based on the TM-score. *Nucleic Acids Res.* **33**, 2302–2309 (2005).
4. Smart, O. S., Neduvelil, J. G., Wang, X., Wallace, B. A. & Sansom, M. S. P. HOLE: A program for the analysis of the pore dimensions of ion channel structural models. *J. Mol. Graph.* **14**, 354–360 (1996).
5. Farci, D., Graça, A. T., Iesu, L., de Sanctis, D. & Piano, D. The SDBC is active in quenching oxidative conditions and bridges the cell envelope layers in Deinococcus radiodurans. *J. Biol. Chem.* **299**, 102784 (2023).
6. Rodríguez, D. D. *et al.* Crystallographic ab initio protein structure solution below atomic resolution. *Nat. Methods 2009 69* **6**, 651–653 (2009).
7. Miroux, B. & Walker, J. E. Over-production of Proteins inEscherichia coli: Mutant Hosts that Allow Synthesis of some Membrane Proteins and Globular Proteins at High Levels. *J. Mol. Biol.* **260**, 289–298 (1996).
8. Ferrières, L. *et al.* Silent mischief: bacteriophage Mu insertions contaminate products of Escherichia coli random mutagenesis performed using suicidal transposon delivery plasmids mobilized by broad-host-range RP4 conjugative machinery. *J. Bacteriol.* **192**, 6418–6427 (2010).
9. Knapp, S. *et al.* Natural Competence Is Common among Clinical Isolates of Veillonella parvula and Is Useful for Genetic Manipulation of This Key Member of the Oral Microbiome. *Front. Cell. Infect. Microbiol.* **7**, (2017).
10. Witwinowski, J. *et al.* An ancient divide in outer membrane tethering systems in bacteria suggests a mechanism for the diderm-to-monoderm transition. *Nat. Microbiol.* **7**, 411–422 (2022).
11. Guzman, L. M., Belin, D., Carson, M. J. & Beckwith, J. Tight regulation, modulation, and high-level expression by vectors containing the arabinose PBAD promoter. *J. Bacteriol.* **177**, 4121–4130 (1995).
12. Van den Berg, B. *et al.* X-ray structure of a protein-conducting channel. *Nature* **427**, 36–44 (2004).
13. Fagan, R. P. & Fairweather, N. F. Clostridium difficile Has Two Parallel and Essential Sec Secretion Systems. *J. Biol. Chem.* **286**, 27483–27493 (2011).
