# Supplementary material for: Dual function of OmpM as outer membrane tether and nutrient uptake channel in diderm Firmicutes
Source: Nat Commun. 2023 Nov 6;14:7152. doi: 10.1038/s41467-023-42601-y (PMC10628300; doi:10.1038/s41467-023-42601-y)
Supplement: Supplementary file 3 — Description of Additional Supplementary Files [file 41467_2023_42601_MOESM3_ESM.pdf]

**Description of Additional Supplementary Files**

File Name: Supplementary Movie 1
Description: All-atom MD simulation of the native VpOmpM1 structure with the grafted AlphaFold2-predicted stalk domain over 1 µs. Left, view parallel to the OM plane; right, view from the periplasm towards the OM. N.B. only the coiled coil part of the stalk is shown.

File Name: Supplementary Movie 2
Description: All-atom MD simulation of the compact structure predicted by AlphaFold2.

File Name: Supplementary Movie 3
Description: A replicate of the all-atom MD simulation of the native VpOmpM1 structure with the grafted AlphaFold2-predicted stalk domain, in which the SLH domain of one protomer unfolds and contacts the coiled coil.
