# Supplementary material for: Dual function of OmpM as outer membrane tether and nutrient uptake channel in diderm Firmicutes
Source: Nat Commun. 2023 Nov 6;14:7152. doi: 10.1038/s41467-023-42601-y (PMC10628300; doi:10.1038/s41467-023-42601-y)
Supplement: Supplementary file 7 — Reporting Summary [file 41467_2023_42601_MOESM7_ESM.pdf]

nature portfolio

Corresponding author(s): Bert van den Berg

Last updated by author(s): 06/10/2023

Reporting Summary

Nature Portfolio wishes to improve the reproducibility of the work that we publish. This form provides structure for consistency and transparency in reporting. For further information on Nature Portfolio policies, see our Editorial Policies and the Editorial Policy Checklist.

Statistics

For all statistical analyses, confirm that the following items are present in the figure legend, table legend, main text, or Methods section.

n/a	Confirmed	
☐	☒	The exact sample size (n) for each experimental group/condition, given as a discrete number and unit of measurement
☐	☒	A statement on whether measurements were taken from distinct samples or whether the same sample was measured repeatedly
☒	☐	The statistical test(s) used AND whether they are one- or two-sided<br>*Only common tests should be described solely by name; describe more complex techniques in the Methods section.*
☒	☐	A description of all covariates tested
☒	☐	A description of any assumptions or corrections, such as tests of normality and adjustment for multiple comparisons
☐	☒	A full description of the statistical parameters including central tendency (e.g. means) or other basic estimates (e.g. regression coefficient) AND variation (e.g. standard deviation) or associated estimates of uncertainty (e.g. confidence intervals)
☒	☐	For null hypothesis testing, the test statistic (e.g. F, t, r) with confidence intervals, effect sizes, degrees of freedom and P value noted<br>*Give P values as exact values whenever suitable.*
☒	☐	For Bayesian analysis, information on the choice of priors and Markov chain Monte Carlo settings
☒	☐	For hierarchical and complex designs, identification of the appropriate level for tests and full reporting of outcomes
☒	☐	Estimates of effect sizes (e.g. Cohen's d, Pearson's r), indicating how they were calculated

Our web collection on statistics for biologists contains articles on many of the points above.

Software and code

Policy information about availability of computer code

Data collection	Cryo-EM:<br>EPU<br><br>Electrophysiology:<br>Clampex
Data analysis	Cryo-EM:<br>cryoSPARC v3.3.2<br>CCPEM v1.6 - program Buccaneer<br>Phenix v1.20.1-4487<br>Coot v0.9.8.8<br>UCSF ChimeraX v1.5<br><br>Crystallography:<br>XIA2<br>Aimless<br>Pointless<br>CCP4i2 v1.1.0 (revision 6539) - programs Arcimboldo, Buccaneer<br>Phenix v1.20.1-4487

Coot v0.9.8.8
UCSF ChimeraX v1.5
Electrophysiology:
Clampfit

Bioinformatics:
AlphaFold v2
ConSurf (web server)
DALI (web server)

Molecular dynamics:
CHARMM-GUI Membrane Builder
CHARMM36m
TIP3P
GROMACS v2021.2
LINCS
MDAnalysis utilities
VMD v1.9.4a51
Molywood v0.22

Liposome swelling assay:
Microsoft Excel 365
RStudio v2022.07.01+554

For manuscripts utilizing custom algorithms or software that are central to the research but not yet described in published literature, software must be made available to editors and reviewers. We strongly encourage code deposition in a community repository (e.g. GitHub). See the Nature Portfolio guidelines for submitting code & software for further information.

Data

Policy information about availability of data

All manuscripts must include a data availability statement. This statement should provide the following information, where applicable:

- Accession codes, unique identifiers, or web links for publicly available datasets
- A description of any restrictions on data availability
- For clinical datasets or third party data, please ensure that the statement adheres to our policy

Electron microscopy maps have been deposited in the Electron Microscopy Data Bank with the accession codes EMD-16328 [https://www.ebi.ac.uk/pdbe/entry/emdb/EMD-16328] (VpOmpM1 from E. coli in C1), EMD-16333 [https://www.ebi.ac.uk/pdbe/entry/emdb/EMD-16333] (VpOmpM1 from E. coli in C3) and EMD-16332 [https://www.ebi.ac.uk/pdbe/entry/emdb/EMD-16332] (VpOmpM1 from V. parvula in C1). Atomic coordinates have been deposited in the Protein Data Bank under accession codes 8BYM [https://doi.org/10.2210/pdb8BYM/pdb] (VpOmpM1 from E. coli in C1), 8BYT [https://doi.org/10.2210/pdb8BYT/pdb] (VpOmpM1 from E. coli in C3), 8BYS [https://doi.org/10.2210/pdb8BYS/pdb] (VpOmpM1 from V. parvula in C1), and 8BZ2 [https://doi.org/10.2210/pdb8BZ2/pdb] (VpOmpM1 stalk crystal structure). The following atomic coordinates used for comparison purposes were downloaded from the Protein Data Bank: 3PYW [https://doi.org/10.2210/pdb3PYW/pdb], 6CWH [https://doi.org/10.2210/pdb6CWH/pdb], 3POQ [https://doi.org/10.2210/pdb3POQ/pdb], 5F7L [https://doi.org/10.2210/pdb5F7L/pdb], 2OMF [https://doi.org/10.2210/pdb2OMF/pdb], 2ZFG [https://doi.org/10.2210/pdb2ZFG/pdb], 8AGD [https://doi.org/10.2210/pdb8AGDpdb]. Trajectories and run input files for the Molecular Dynamics simulations are available on Zenodo (https://doi.org/10.5281/zenodo.8239075). Source data are available. Other data presented in this paper, constructs and strains are available on reasonable request.

Research involving human participants, their data, or biological material

Policy information about studies with human participants or human data. See also policy information about sex, gender (identity/presentation), and sexual orientation and race, ethnicity and racism.

Reporting on sex and gender	N/A
Reporting on race, ethnicity, or other socially relevant groupings	N/A
Population characteristics	N/A
Recruitment	N/A
Ethics oversight	N/A

Note that full information on the approval of the study protocol must also be provided in the manuscript.

Field-specific reporting

Please select the one below that is the best fit for your research. If you are not sure, read the appropriate sections before making your selection.

☒ Life sciences ☐ Behavioural & social sciences ☐ Ecological, evolutionary & environmental sciences

For a reference copy of the document with all sections, see nature.com/documents/nr-reporting-summary-flat.pdf

Life sciences study design

All studies must disclose on these points even when the disclosure is negative.

Sample size	No statistical methods were used to predetermine sample sizes.<br>For cryo-EM, the collected datasets contained enough particles for structure determination.<br>All other experiments were performed at least three times to ensure reproducibility.
Data exclusions	Cryo-EM movies with unacceptable CTF parameters, average intensity and relative ice thickness were excluded from data analysis. Particle images that did not yield good 2D class averages or 3D reconstructions were excluded from single particle analysis.<br>In liposome swelling assays if the linear rate of swelling was missed due to starting the measurement too late, the data were excluded from analysis and another replicate was performed.<br>Non-physiological currents observed in electrophysiology recordings, e.g. excessively drifting or excessively large currents, were excluded from analysis.
Replication	Structure determination experiments were not repeated, as is standard practice in the field.<br>Liposome swelling assays were done in technical triplicates, as independent replicates are not possible due to unpredictable batch-to-batch variability of proteoliposome preparations. Similar trends were observed in at least three different proteoliposome preparations.<br>Electrophysiology experiments were repeated as many times as feasible to ensure reproducibility. Different amounts of data were collected for the three proteins due to their different propensities for bilayer insertion.
Randomization	Particle stacks were randomly split by cryoSPARC into two independent sets during 3D volume reconstruction in single particle analysis.<br>For all other experiments, randomization was not possible.
Blinding	Knowing the identities of the samples was required for carrying out the analyses, and no blinding was performed as part of data analysis.

Reporting for specific materials, systems and methods

We require information from authors about some types of materials, experimental systems and methods used in many studies. Here, indicate whether each material, system or method listed is relevant to your study. If you are not sure if a list item applies to your research, read the appropriate section before selecting a response.

Materials & experimental systems

n/a	Involved in the study
☒	☐ Antibodies
☒	☐ Eukaryotic cell lines
☒	☐ Palaeontology and archaeology
☒	☐ Animals and other organisms
☒	☐ Clinical data
☒	☐ Dual use research of concern
☒	☐ Plants

Methods

n/a	Involved in the study
☒	☐ ChIP-seq
☒	☐ Flow cytometry
☒	☐ MRI-based neuroimaging
