# Supplementary figures and images for: Dual function of OmpM as outer membrane tether and nutrient uptake channel in diderm Firmicutes
Source: Nat Commun. 2023 Nov 6;14:7152. doi: 10.1038/s41467-023-42601-y (PMC10628300; doi:10.1038/s41467-023-42601-y)

**Figure 3a**

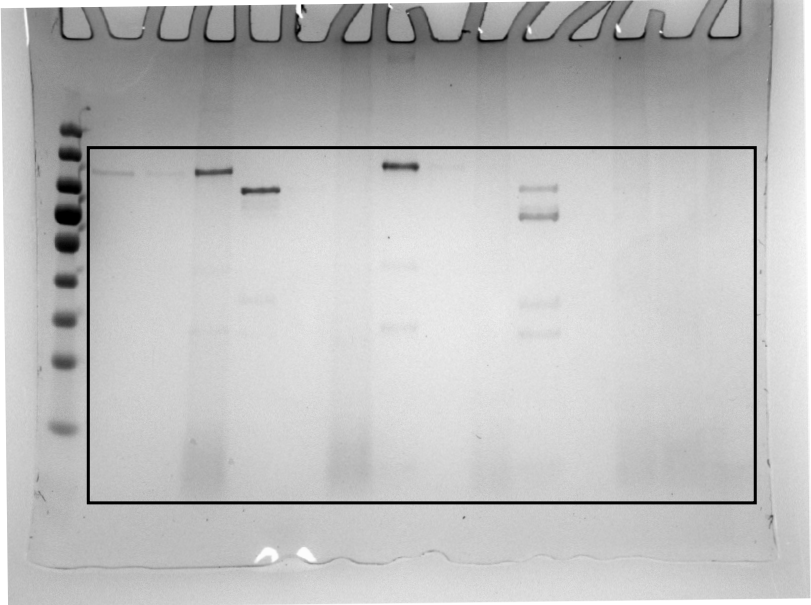

**Figure 4f**

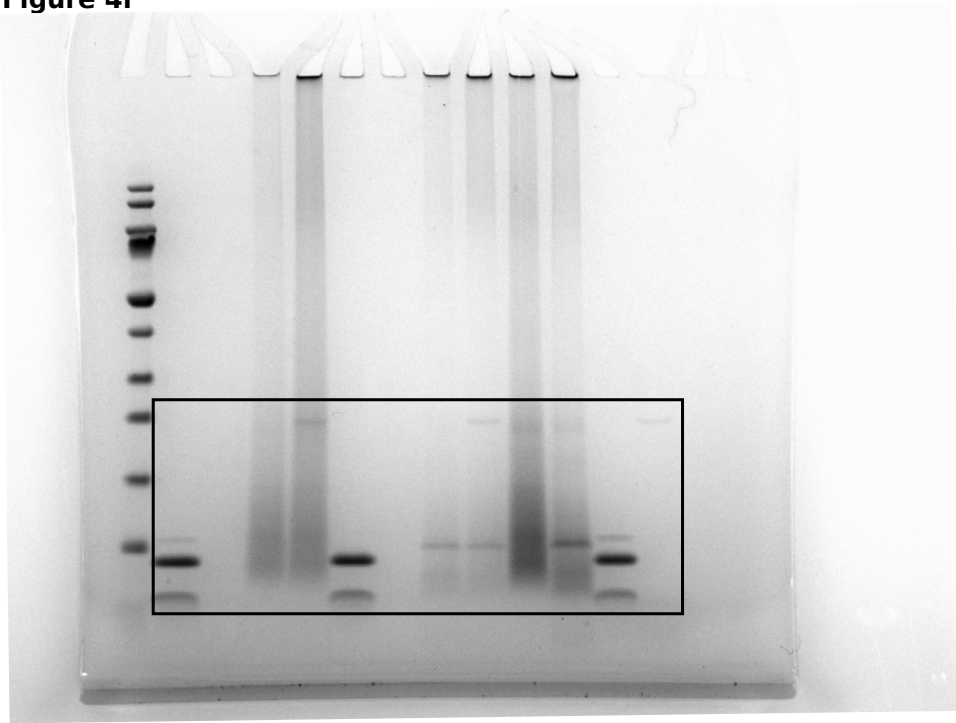

Supplement: Supplementary file 8 — Source Data [file 41467_2023_42601_MOESM8_ESM.zip › SourceData_uncropped_gels.pdf]
